# The effects of neoadjuvant chemoradiotherapy and an in-hospital exercise training programme on physical fitness and quality of life in locally advanced rectal cancer patients: a randomised controlled trial (The EMPOWER Trial)

**DOI:** 10.1186/s13741-021-00190-8

**Published:** 2021-06-22

**Authors:** Lisa Loughney, Malcolm A. West, Helen Moyses, Andrew Bates, Graham J. Kemp, Lesley Hawkins, Judit Varkonyi-Sepp, Shaunna Burke, Christopher P. Barben, Peter M. Calverley, Trevor Cox, Daniel H. Palmer, Michael G. Mythen, Michael P. W. Grocott, Sandy Jack, on behalf of the Fit4Surgery group

**Affiliations:** 1 Anaesthesia and Critical Care Research Area, NIHR Biomedical Research Centre, University Hospital Southampton NHS Foundation Trust, Road, Southampton, UK; 2 Integrative Physiology and Critical Illness Group, Clinical and Experimental Sciences, Faculty of Medicine, University of Southampton, Southampton, UK; 3 ExWell Medical, Dublin, Ireland; 4 Cancer Sciences Unit, Faculty of Medicine, University of Southampton, Southampton, UK; 5 Departments of Anaesthesia and Critical Care, Royal Bournemouth NHS Foundation Trust, Bournemouth, UK; 6 Department of Musculoskeletal Biology and MRC – Arthritis Research UK Centre for Integrated research into Musculoskeletal Ageing (CIMA), Faculty of Health and Life Sciences, University of Liverpool, Liverpool, UK; 7 Faculty of Biological Sciences, School of Biomedical Sciences, University of Leeds, Leeds, UK; 8 Department of Colorectal Surgery, Aintree University Hospitals NHS Foundation Trust, Liverpool, UK; 9 Institute of Ageing and Chronic Disease, University of Liverpool, Liverpool, UK; 10 Cancer Research UK Liverpool Cancer Trials Unit, University of Liverpool, Liverpool, UK; 11 Institute of Translational Medicine, University of Liverpool, Liverpool, UK; 12 Anaesthesia and Critical Care, University College London, London, UK

**Keywords:** Exercise prehabilitation, Neoadjuvant cancer treatment, Physical fitness, Rectal cancer, Surgery

## Abstract

**Background:**

The EMPOWER trial aimed to assess the effects of a 9-week exercise prehabilitation programme on physical fitness compared with a usual care control group. Secondary aims were to investigate the effect of (1) the exercise prehabilitation programme on psychological health; and (2) neoadjuvant chemoradiotherapy (NCRT) on physical fitness and psychological health.

**Methods:**

Between October 2013 and December 2016, adults with locally advanced rectal cancer undergoing standardised NCRT and surgery were recruited to a multi-centre trial. Patients underwent cardiopulmonary exercise testing (CPET) and completed HRQoL questionnaires (EORTC-QLQ-C30 and EQ-5D-5L) pre-NCRT and post-NCRT (week 0/baseline). At week 0, patients were randomised to exercise prehabilitation or usual care (no intervention). CPET and HRQoL questionnaires were assessed at week 0, 3, 6 and 9, whilst semi-structured interviews were assessed at week 0 and week 9. Changes in oxygen uptake at anaerobic threshold (VO_2_ at AT (ml kg^−1^ min^−1^)) between groups were compared using linear mixed modelling.

**Results:**

Thirty-eight patients were recruited, mean age 64 (10.4) years. Of the 38 patients, 33 were randomised: 16 to usual care and 17 to exercise prehabilitation (26 males and 7 females). Exercise prehabilitation significantly improved VO_2_ at AT at week 9 compared to the usual care. The change from baseline to week 9, when adjusted for baseline, between the randomised groups was + 2.9 ml kg ^−1^ min ^−1^; (95% CI 0.8 to 5.1), *p* = 0.011.

**Conclusion:**

A 9-week exercise prehabilitation programme significantly improved fitness following NCRT. These findings have informed the WesFit trial (NCT03509428) which is investigating the effects of community-based multimodal prehabilitation before cancer surgery.

**Trial registration:**

ClinicalTrials.gov NCT01914068. Registered 1 August 2013.

**Supplementary Information:**

The online version contains supplementary material available at 10.1186/s13741-021-00190-8.

## Background

The standard multi-modal treatment with curative intent for magnetic resonance imaging (MRI)-defined, resection margin-threatened, locally advanced rectal cancer is long course neoadjuvant chemoradiotherapy (NCRT) followed by a waiting period before surgery (NICE 2020). However, some centres in the UK prefer a multi-modal treatment pathway of systemic neoadjuvant chemotherapy prior to NCRT followed by a waiting period before surgery or even short course NCRT with a much shorter wait period before surgery. Although such multimodal treatment pathways have improved cancer outcomes mainly by reducing local recurrence rates (Fanke et al. 2018; Pucciarelli et al. 2009), they are associated with a risk of toxicity and a significant risk of morbidity and mortality (Pearse et al. 2012).

There is strong evidence that cardiopulmonary exercise testing (CPET) predicts post-operative morbidity and mortality in several surgical groups (Carlisle and Swart 2007; Lee et al. 2018; Moran et al. 2016; Snowden et al. 2010; Wijeysundera et al. 2018; Wilson et al. 2010). In people with newly diagnosed rectal cancer, NCRT prior to surgery has been shown to significantly reduce physical fitness, as measured by CPET, and this reduction has been linked to adverse post-operative outcome (West et al. 2014a). The standard waiting period between completion of NCRT and surgery is generally at least 8 weeks (Du et al. 2017) (or in some National Health Service (NHS) hospitals up to 14 weeks) and therefore represents a unique opportunity to intervene with exercise prehabilitation. In this area of research, early evidence demonstrates the feasibility and preliminary effectiveness of exercise training (unimodal prehabilitation delivered in varied formats) for people with rectal cancer scheduled for NCRT and surgery (Heldens et al. 2016; Morielli et al. 2016a; Morielli et al. 2016b; Morielli et al. 2018a; Moug et al. 2019; Singh et al. 2018; West et al. 2014b). To date, however, no exercise RCTs have been reported on both physical and psychological health measures in people with rectal cancer (although the EXERT trial is currently recruiting) (Morielli et al. 2018b) or indeed any patient group scheduled for a multimodal treatment pathway (Loughney et al. 2018).

The primary aim of the EMPOWER trial was to assess the effects of a 9-week exercise prehabilitation programme on physical fitness, compared with a usual care control group. Secondary aims were to investigate the effects of (1) the exercise prehabilitation on psychological health and (2) NCRT on physical fitness and psychological health. Other exploratory outcomes included physical activity (PA), post-operative morbidity, cancer staging and safety.

## Methods

### Setting and participants

The methodology for the EMPOWER trial has been published elsewhere (Loughney et al. 2016), registered with ClinicalTrials.gov NCT01914068 (registered 1 August 2013, https://clinicaltrials.gov/ct2/show/NCT01914068). EMPOWER is a multi-centre trial which was conducted in five UK NHS hospitals. Briefly, this was a parallel group (exercise prehabilitation vs. usual care control), observer blinded (blind to CPET data, group allocation and outcomes), randomised controlled clinical trial. Patients with MRI-defined, circumferential margin-threatened, potentially curable, locally advanced rectal cancer undergoing both NCRT and elective surgery were recruited. All potentially eligible patients were identified at multi-disciplinary meetings and approached at an outpatient appointment. Patients were contacted by telephone to provide additional information about the trial. Written informed consent was provided at the pre-NCRT visit. Recruitment took place between 2013 and 2016.

### Neoadjuvant cancer treatment

Neoadjuvant cancer treatments varied depending on the hospital. The ‘standard’ chemoradiotherapy programme was 45 Gy in 25 fractions on weekdays using a 3D conformal technique with CT guidance and oral capecitabine 900 mg m^−2^ twice daily on radiotherapy days. Although the role of neoadjuvant systemic chemotherapy is still under investigation (Habr-Gama et al. 2009; Nilsson et al. 2013; Wyrwicz et al. 2016), during recruitment to the EMPOWER trial, University Hospital Southampton NHS Foundation Trust and Royal Bournemouth Christchurch Hospitals adopted systemic neoadjuvant chemotherapy prior to chemoradiotherapy as a standard treatment. Eight participants were scheduled for this treatment which consisted of oxaliplatin 130 mg m^−2^ intravenously on day 1, oral capecitabine 1000 mg m^−2^ (days 2–15) given in a 3 weekly cycle × 4 cycles over 12 weeks, followed the standard chemoradiotherapy programme as described above.

### Randomisation

Following neoadjuvant CRT (week 0), patients were allocated to the in-hospital exercise training group or usual care control group. Patients were randomised (1:1) to either an in-hospital exercise prehabilitation programme or usual care control group using the Trans European Network for patient randomisation in clinical trials system (TENAELA System) and concealed by the research team.

### Interventions

#### Usual care control group

The usual care control group received routine care throughout their cancer pathway from diagnosis to surgical resection (no exercise intervention).

#### Exercise prehabilitation intervention group

Participants started their exercise training on the first week after NCRT (week 0) in their treating hospital and were scheduled to attend 3 sessions/week for 9 weeks. The delivery of the exercise programme is described elsewhere (Loughney et al. 2016) and in Supplementary Appendix 1 according to the Consensus on Exercise Reporting Template guidelines (Slade et al. 2016).

### Exercise training adherence

Exercise adherence was assessed by number of sessions attended compared to number of planned sessions (i.e. 3 sessions/week × 9 weeks)

### Outcome measurements

All patients were assessed pre-NCRT, after completion of NCRT (week 0/baseline) and at weeks 3, 6 and 9.

### Primary outcome

#### Physical fitness

The CPET-derived variable, oxygen uptake at anaerobic threshold (VO_2_ at AT (ml kg^−1^ min ^−1^), was used to assess physical fitness at all time points. CPET data was reported according to the Perioperative Exercise Testing and Training Society (POETTS) guidelines (which were co-developed by authors SJ and MPG) (Levett et al. 2018). The CPET protocol was identical at each hospital (described elsewhere (Loughney et al. 2016)) using the same software and the same metabolic cart (Geratherm Respiratory BmbH, Love Medical Ltd.). Final CPET analysis was conducted using the modified V-Slope method by two independent physiological assessors blinded to group allocation, each other and clinical outcomes. Any discrepancies (variance by more than 5% in VO_2_ at AT between the first two assessors) were resolved by a third assessor.

### Secondary outcome

#### Health-related quality of life (HRQoL)

HRQoL was assessed by the following:

1. Semi-structured interviews: at week 0 (following NCRT) and at week 9 (prior to surgery) to explore patients’ perspectives of their HRQoL using methods previously piloted by our group (Fit-4-Surgery) (Burke et al. 2013).
2. Questionnaires: the European Organization for Research and Treatment of Cancer Quality of Life Questionnaire (EORTC QLQ-C30; 30 items) (Aaronson et al. 1993) and EQ-5D-5L (Rabin and de Charro 2001) were administered at the same time-points as CPET.

### Exploratory outcome measures

#### Physical activity

Daily physical activity was measured using a multi-sensory accelerometer (SenseWear Pro® armband (Model MF-SW, display model DD100); BodyMedia, Inc., Pittsburgh, PA, USA). Monitors were issued to participants at their CPET visits. Participants were asked to wear the monitor for three consecutive days and nights (72 h of continuous data). Outcome measures of interest for physical activity were daily step count and sleep efficiency.

#### Post-operative morbidity survey (POMS)

Post-operative outcome was objectively recorded using POMS (Grocott et al. 2007) at days 3, 5, 8 and 15.

#### Cancer staging

Rectal cancer tumour regression was quantified by MRI assessment using the MRI-based T-staging tumour regression grade (ymrTRG) utilizing the MERCURY group protocol (Patel et al. 2011, 2012) and the histopathological tumour regression grading (ypTRG) (Dworak et al. 1997).

#### Safety

All adverse events to CPET or the exercise prehabilitation programme were recorded in the relevant case report forms.

### Data analysis

#### Sample size calculation

A sample of 46 patients was required to detect a difference between groups of 2.0 ml kg^−1^ min^−1^ VO_2_ at AT using a two-sample *t* test at the 5% significance level with 90% power. This was based on a standard deviation of the change in VO_2_ at AT of 1.8 ml kg^−1^ min^−1^ and was inflated to allow for 20% patient drop-out (Kothmann et al. 2009).

### Statistical analyses

Descriptive analyses were carried out to summarise patients’ characteristics. Continuous variables are reported as mean (SD) or median and inter-quartile range (IQR), depending on distribution, and categorical variables as frequency (%). The Shapiro-Wilk test for normality of distributions was applied. Estimates obtained from statistical tests are reported with 95% CI. The effect of NCRT on physical fitness was assessed using a paired *t* test. The primary analysis was intention to treat. For the primary outcome of VO_2_ AT, all randomised participants with baseline (week 0) were included. Multiple imputation was used to handle any missing outcome data for the primary endpoint.

A linear regression model (using age and gender) was used to investigate the effect of the exercise prehabilitation intervention on physical fitness and a 95% confidence interval on the mean difference between the two groups for both unadjusted and baseline-adjusted models. In order to assess the sensitivity of results to covariates, a further ‘adjusted’ effect was calculated using linear regression with age and gender as covariates (Belle 2002). Linear mixed modelling was employed to compare fitness over all time points.

Linear regression models were used to compare overall HRQoL scores between groups and EORTC QLQ-30 sub-scales, and multilevel mixed effects ordinal regression was used to assess EQ-5D-5L sub-scales. Interview data were analyzed using a thematic framework approach (Burke et al. 2013).

The effect of exercise prehabilitation on physical activity was analysed in the same way as physical fitness. Cancer staging was reported as tumour, node metastasis version 5 (TNM) staging with T3 sub-staging, Response Evaluation Criteria in Solid Tumours (RECIST) and ymrTRG (Glimelius et al. 2010). Pathological outcomes were graded according to the pathological tumour regression grading (ypTRG) and ypTNM. In our protocol paper (Loughney et al. 2016), we stated that we would conduct univariate logistic regression analysis to analyse the association between demographic variables (patient age and sex), MRI parameters, and pathologic tumour response, to enable the calculation of odds ratios for the probability of an unfavourable pathological outcome, and to analyse the sensitivity, specificity, positive and negative likelihood ratios. However, because of low number of participants in each group who completed NCRT and surgery, this analysis was not performed.

## Results

**Fig. 1:**
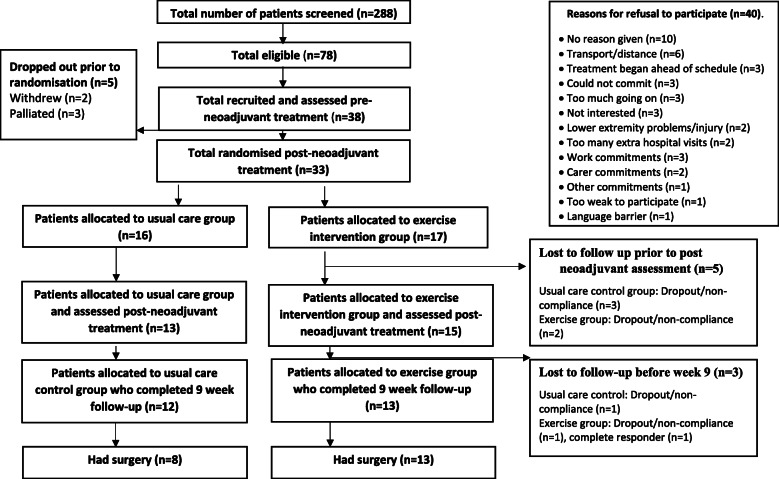
Screening and recruitment algorithm

Between October 2013 and December 2016, 78 patients met the inclusion criteria and 38 agreed to participate (Fig. 1). Of the 38 patients recruited, 15 were from University Hospital Southampton NHS Foundation Trust, 12 from Aintree University Hospitals NHS Foundation Trust, four from South Tees Hospitals NHS Foundation Trust and seven from Royal Bournemouth Christchurch Hospitals. Thirty-three were randomised (16 to the usual care control group and 17 to the exercise prehabilitation group), 25 completed the follow-up assessment at week 9 and 21 underwent surgery. Patient characteristics are presented in Table 1. Sixteen participants received neoadjuvant chemotherapy prior to the standard NCRT regimen whilst the remainder received the standard NCRT regimen. Adherence to the 9-week exercise programme was 91% and the exercise programme was adapted and progressed every 3 weeks for all participants based on repeat CPET assessments.

**Table 1:** Patient characteristics

	Exercise (***n*** = 17)	Control (***n*** = 16)	Non-randomised (***n*** = 5)
**Age (years)**	64 (14)^a^	57 (10)^a^	68 (7)^a^
**Gender (% male)**	14 (82%)	12 (75%)	4 (80%)
**Smoking (%) current**	4 (24%)	2 (13%)	1 (20%)
**Body mass index (BMI) (kg/m**^**2**^**)**	26 (4) ^a^	26 (3) ^a^	24 (4) ^a^
**Past medical history**			
Heart failure	0 (0%)	0 (0%)	0 (0%)
Diabetes	2 (12%)	1 (6%)	0 (0%)
Ischemic heart disease	0 (0%)	0 (0%)	0 (0%)
Cerebrovascular disease	0 (0%)	0 (0%)	0 (0%)
**Cancer staging**			
Tumor			
T2	1 (6%)	2 (13%)	0 (0%)
T3/T3a	9 (53%)	8 (50%)	1 (20%)
T3b	2 (12%)	2 (13%)	0 (0%)
T3c	2 (12%)	1 (6%)	0 (0%)
T3d	0 (0%)	1 (6%)	0 (0%)
T4	3 (18%)	2 (13%)	3 (60%)
Missing	0 (0%)	0 (0%)	1 (20%)
Node			
N0	4 (24%)	4 (25%)	0 (0%)
N1	7 (41%)	7 (44%)	3 (60%)
N2	6 (35%)	5 (31%)	1 (20%)
Missing	0 (0%)	0 (0%)	1 (20%)
Metastases			
M0	16 (94%)	16(100%)	4 (80%)
M1	1 (6%)	0 (0%)	0 (0%)
Missing	0 (0%)	0 (0%)	1 (20%)
**Operation**			
Abdomino-perineal excision	4 (24%)	3 (19%)	0 (0%)
Anterior resection	8 (47%)	3 (19%)	0 (0%)
Central pelvic exenteration	0 (0%)	2 (13%)	0 (0%)
Hartmann’s and liver resection	1 (6%)	0 (0%)	0 (0%)
No surgery (complete responder)	1 (6%)	3 (19%)	0 (0%)
No surgery (disease progression)	1 (6%)	1 (6%)	3 (60%)
No surgery (drop-out/withdrawn)	2 (12%)	4 (25%)	2 (40%)

Cancer staging is reported using TNM classification (V.5, 1997) with sub-classifications. Central pelvic exenteration included excision of rectum and pelvic side wall ± lymphadenectomy

^a^All data are presented as *n* (%) mean except for age and BMI which are mean (SD)

### Primary outcome

#### Physical fitness

The change in VO_2_ at AT from baseline to week 9, adjusted for baseline, between treatment groups was + 2.9 ml kg^−1^ min^−1^; (95%CI 0.8, 5.1), *p* = 0.011 (Table 2) (when unadjusted for baseline was + 2.8 (95%CI 0.6, 5.0), *p* = 0.014). Sensitivity analysis between treatment groups for a further adjusted effect using linear regression for age and gender was + 3.4 ml kg^−1^ min ^−1^; (95%CI 1.1, 5.7), *p* = 0.005.

**Table 2:** Physical fitness at week 0 and week 9 between exercise and usual care control groups

Physical fitness	Week 0, ***n***	Week 9, ***n***	Difference in endpoint week 9 (95% CI), ***P*** value
**Primary outcome:** VO_2_ at AT (ml kg^−1^ min ^−1^)			
*Exercise*	11.6 (3.4), 15	15.0 (4.2), 13	2.9 (0.8, 5.1), 0.011
*Usual care control*	10.8 (2.5), 13	11.5 (2.5), 12	

Data are presented as mean (SD)

*Abbreviations*: *VO*_*2*_ *at AT* oxygen uptake at anaerobic threshold, *n* sample of patients assessed

*P < 0.05 was taken as statistically significant. P value adjusted for baseline following linear regression with the difference between week 9 and week 0

**Fig. 2:**
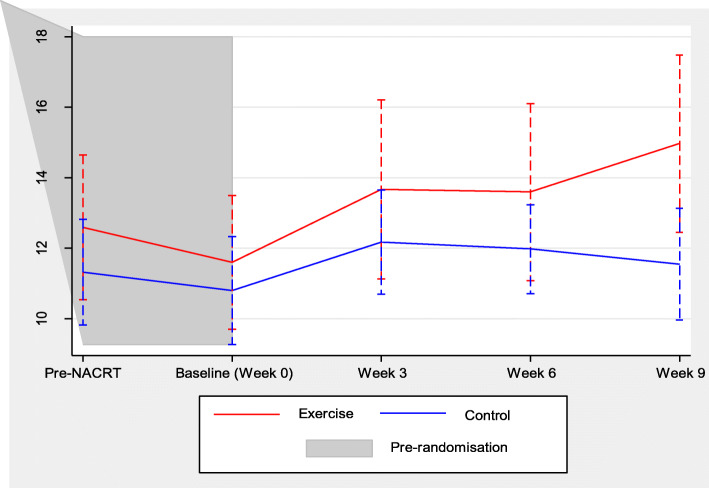
Changes in oxygen uptake at lactate threshold (ml kg^−1^ min ^−1^) throughout the entire cancer journey: pre-NCRT, post-NCRT (baseline/week 0), week 3, 6 and 9 between the exercise group and the usual care control group

Changes in VO_2_ at AT throughout the entire cancer journey: pre-NCRT, post-NCRT (week 0), week 3, 6 and 9 between the treatment groups is graphically presented in Fig. 2. Changes in all CPET variables between the groups over the 9-week study period are presented in Supplementary Appendix 2, Table S1 and individual graphical plots for changes in VO_2_ at AT over the 9-week study period are presented in Appendix 3.

### Secondary outcomes

From pre- to post-NCRT, VO_2_ at AT significantly decreased in all participants: − 1.3 ml kg^−1^ min^−1^; (95%CI 0.4, 2.3), *p* = 0.008 (Table 3). Changes in all CPET variables between pre- and post-NCRT are presented in Supplementary Appendix 4, Table S2.

#### HRQoL

1. HRQoL questionnaires

From pre- to post-NCRT, there was a significant reduction in EORTC Global Health Status − 7.1 (95%CI − 0.8, − 13.5), *p* = 0.030 and EQ5D usual activities – 8.6 (95%CI 1.4, 52.8), *p* = 0.020 (Table 3). There were no other significant changes in HRQoL quantitative measurements between the treatment groups over the 9-week study period (Table 4).

(2) Semi-structured interviews

**Table 3:** Physical fitness, health-related quality of life (questionnaires) and physical activity pre- and post-neoadjuvant cancer treatment

	Pre-NCRT, ***n***	Post-NCRT, ***n***	Mean difference (95 % CI)	***P*** value
**Primary outcome:** VO_2_ at AT (ml kg^−1^ min^−1^)	12.6 (3.3), 28	11.2 (3),28	1.3 (0.4, 2.3)	0.008^a^ *
**Secondary outcome: EORTC C30 questionnaire** (scale scores between 0 and 100 (best)				
*Global health status*	70.4 (16.2), 27	63.7 (16.2),28	7.1 (0.8, 13.5)	0.030^a^ *
*Physical functioning*	93.3 (80.0, 100.0), 27	86.7 (73.3, 100).28		0.119^b^
*Emotional functioning*	83.3 (66.7, 91.7), 27	75.0 (66.7, 91.7),28	1.2 (− 4.9, 7.3)	0.740^a^
*Fatigue*	22.2 (11.1, 33.3), 27	33.3 (22.2, 55.6),28	− 16.5 (− 26.5, − 6.4)	0.002^a^ *
*Pain*	16.7 (0.0, 33.3), 27	16.7 (0, 33.3),28	− 9.3 (− 20.1, 1.6)	0.092^a^
*Insomnia*	33.3 (0.0, 33.3), 27	33.3 (0, 66.6),28	− 11.1 (− 20.8, − 1.4)	0.026^a^ *
**EQ5D questionnaire:** ***usual activities***	**Pre-NCRT,** ***n***	**Post-NCRT,** ***n***	**Odds ratio (95% CI)**	***P*** **value**
*No problems doing usual activity*	18 (67%), 27	14 (50%),28	8.6 (1.4, 52.8)	0.020^a^ *
*Slight problems doing my usual activity*	7 (26%), 27	8 (28.6%),28		
*Moderate problems doing usual activity*	2 (7.4%), 27	5 (17.9%),28		
*Severe problems doing usual activity*	0 (0%), 27	1 (3.6%),28		
***Self-care***				
*No problems washing or dressing*	26 (96.3%), 27	26 (92.9%),28	2.4 (0.2, 28.4)	0.476^a^
*Slight problems washing or dressing*	1 (3.7%), 27	1 (3.6%),28		
*Moderate problems washing or dressing*	0 (0%), 27	1 (3.6%),28		
***Pain/discomfort***				
*No pain or discomfort*	10 (37.0%), 27	8 (28.6%),28	0.8 (0.3, 2.4)	0.721^a^
*Slight pain or discomfort*	11 (40.7%), 27	15 (53.6%),28		
*Moderate pain or discomfort*	5 (18.5%), 27	5 (18.9%),28		
*Severe pain or discomfort*	1 (3.7%), 27	0 (0%),28		
***Anxiety/depression***				
*Not anxious or depressed*	14 (51.9%), 27	15 (53.6%),28	0.6 (0.2, 2.2)	0.478^a^
*Slightly anxious or depressed*	8 (29.6%), 27	11 (39.3%),28		
*Moderately anxious or depressed*	4 (14.8%), 27	1 (3.6%),28		
*Severely anxious or depressed*	1 (3.7%), 27	1 (3.6%),28		
Health scale (between 0 and 100 (best)	74.8 (16.8), 27	70.9 (14.4),28	4.2 (− 1.4, 9.9)	0.121^a^
**Exploratory outcome: physical activity**				
Step count	5276 (3590, 8648), 22	5641 (3602, 8929),25	84.3 (− 1340.5, 1509.1)	0.904^a^
Sleep efficiency (%)	79.1 (69.6, 85.0), 21	79.1 (67.5, 82.9),25		0.354^b^
PA duration (min/day)	52.5 (28.7, 108.0), 22	70.3 (28, 121),25	− 0.2 (− 20.0, 19.5)	0.982^a^
Energy expenditure (kcal/day)	2258.3 (629.7), 22	2419.8 (593.5),25	− 37.5 (− 199.8, 124.8)	0.636^a^
Sleep duration (min/day)	391.1 (118.7), 22	377.3 (79.6),25	9.6 (− 40.9, 60.1)	0.697^a^
Average METS	1.4 (0.3), 22	1.3 (0.2),25	0.02 (− 0.07, 0.1)	0.593^a^

*Abbreviations*: *VO*_*2*_ *at AT* oxygen uptake at anaerobic threshold, *VO*_*2*_ *at Peak* oxygen uptake at peak exercise, *PA* physical activity, *METS* metabolic equivalent threshold, *n* sample of patients assessed

Note: physical activity data is mean over 3 days

Data are presented as mean (SD), median (IQR) or *n* (%)

*P < 0.05 was taken as statistically significant

^a^*P* value following paired sample *t* tests

^b^*P* value following Wilcoxon signed-rank tests

Post-NCRT, the semi-structured interviews revealed that cancer treatment adversely impacted participants HRQoL. Four main themes (each involving subthemes) were identified: physical ill-being, social problems, behavioural/lifestyle disruptions and psychological ill-being. However, participation in the 9-week exercise prehabilitation programme resulted in positive changes in perceptions in HRQoL. Two main themes (each involving subthemes) were identified: physical well-being and psychological well-being (Supplementary Appendix 5, Table S3).

### Exploratory outcomes

#### Physical activity

From pre- to post-NCRT, there were no significant differences in daily step count, 84.3 (95%CI *−* 1340.5, 1509.1), *p* = 0.904, or for sleep efficiency median (interquartile range) 80.2 (71.3, 85.3) and week 9: 79.1 (67.5, 82.9), *p* = 0.354 (Table 3). Direct comparison between treatment groups found no significant difference between week 9 and week 0 using a linear model adjusted for baseline in daily step count: *−* 46.3 (95%CI *−* 2045, 1952), *p* = 0.877, or sleep efficiency: *−* 3.0 (95%CI *−* 15.2, 9.3), *p* = 0.919. There were no significant differences between week 9 and week 0 for any other physical activity outcomes (Table 4).

**Table 4:** Physical fitness, health-related quality of life (questionnaires) and physical activity at week 0 and week 9 between exercise and usual care control groups

	Exercise		Usual Care Control		***P*** value
	Week 0, ***n***	Week 9, ***n***	Week 0, ***n***	Week 9, ***n***	
**Secondary outcome: EORTC C30 questionnaire** (scale scores between 0 and 100 (best)					
*Global health status*	63.9 (16.6),15	77.8 (13.0),12	63.5 (16.5),13	72 (19.5),11	0.668 ^a^
*Physical functioning*	86.7 (73.3, 100.0),15	100 (86.7, 100.0), 2	86.7 (66.7, 93.3),13	86.7 (86.7, 100),11	0.782^b^
*Emotional functioning*	75.0 (66.7, 100.0),15	95.8 (66.7, 100.0),12	75.0 (66.7, 83.3),13	75.0 (50, 83.3),11	0.132^a^
*Fatigue*	33.3 (11.1, 55.6),15	11.1 (0.0, 33.3),12	33.3 (22.2, 55.6),13	11.1 (0, 33.3),11	0.603^a^
*Pain*	16.7 (0.0, 33.3),15	0 (0.0, 8.3),12	16.7 (16.7, 33.3),13	16.7 (0, 33.3),11	0.708^b^
*Insomnia*	33.3 (0.0, 66.7),15	16.7 (0.0, 33.3),12	33.3 (0.0, 66.7),13	33.3 (0, 33.3),11	0.248^a^
**EQ5D questionnaire:** ***usual activities***					
*No problems doing usual activity*	8 (53%),15	6 (46%),12	9 (75%),13	7 (64%),11	0.630^a^
*Slight problems doing usual activity*	4 (27%),15	4 (31%),12	3 (25%),13	4 (36%),11	
*Moderate problems doing usual activity*	2 (13%),15	3 (23%),12	0 (0%),13	0 (%),11	
*Severe problems doing usual activity*	1 (7%),15	0 (0%),12	0 (0%),13	0 (0%),11	
***Self-care***					
*No problems washing/dressing*	15 (100%),15	11 (85%),12	12 (100%),13	10 (91%),11	0.491
*Slight problems washing/dressing*	0 (0%),15	1 (8%),12	0 (0%),13	1 (9%),11	
*Moderate problems washing/dressing*	0 (0%),15	1 (8%),12	0 (%),13	0 (0%),11	
***Pain/discomfort***					
*No pain or discomfort*	4 (27%),15	4 (31%),12	9 (75%),13	5 (45%),11	0.512^a^
*Slight pain or discomfort*	9 (60%),15	6 (46%),12	2 (17%),13	4 (36%),11	
*Moderate pain or discomfort*	2 (13%),15	3 (23%),12	1 (8%),13	2 (18%),11	
***Anxiety/depression***					
*Not anxious or depressed*	10 (67%),15	5 (38%),12	9 (75%),13	5 (45%),11	0.111^a^
*Slightly anxious or depressed*	4 (27%),15	7 (54%),12	3 (25%),13	4 (36%),11	
Moderately anxious or depressed	1 (7%),15	0 (0%),12	0 (0%),13	1 (9%),11	
Severely anxious or depressed	0 (0%),15	1 (8%),12	0 (0%),13	0 (0%),11	
Extremely anxious or depressed	0 (0%),15	0 (%),12	0 (0%),13	1 (9%),11	
Health scale (between 0 and 100 (best)	69.6 (14),15	72.4 (15.2),12	78.8 (14),13	75.1 (20.3),11	0.040^a^ *
**Exploratory outcome: physical activity**					
Step count	7058 (4981),12	7023 (5562),11	6321 (3456),13	6749 (2959),12	0.877^a^
Sleep efficiency (%)	74.5 (8.3),12	72.1 (13.2),11	75.7 (10.5),13	74.4 (12.8),12	0.919^a^
Energy expenditure (kcal/day)	2467 (676), 12	2326 (639),11	2376 (530),13	2424 (559),12	0.145^a^
PA duration (min/day)	96.9 (79.5),12	89.5 (81.8),11	70.8 (51.2),13	81.2 (43.8),12	0.313^a^
Sleep duration (min/day)	380.4 (75.7),12	373.9 (82.8),11	374.5 (86.1),13	361.6 (98.5),12	0.803^a^
Average METS	1.4 (0.2),12	1.4 (0.2),11	1.3 (0.2),13	1.3 (0.2),12	0.525^a^

Note: physical activity data is mean data over 3 days

Data are presented as mean (SD) or median (IQR) except EQ5D data which is presented as *n* (%)

*Abbreviations*: *PA* physical activity, *METS* metabolic equivalent threshold, *n* sample of patients assessed

*P < 0.05 was taken as statistically significant

^a^*P* value following linear regression with the difference between week 9 and 0 adjusted for baseline

^b^*P* value following Wilcoxon Mann-Whitney test

#### Post-operative morbidity survey and cancer staging

Post-operative morbidity survey data for both treatment groups are reported descriptively in Supplementary Appendix 6, Table S4 (no formal analyses were conducted).

The response of NCRT for both treatment groups are reported descriptively in Supplementary Appendix 7 Table S5. Four participants had a complete clinical response and did not undergo any surgery.

#### Safety

There were no serious adverse events. There was one adverse event attributable to exercise training where a participant sustained a pre-syncope episode following one of the exercise sessions. The participant was reviewed by the hospital medical team and by the patient’s own general practitioner, with no pause in exercise training.

## Discussion

The EMPOWER trial demonstrates physical fitness levels and HRQoL were significantly reduced following NCRT in people with locally advanced rectal cancer. However, a 9-week exercise prehabilitation programme offered in local treating hospitals initiated following completion of NCRT significantly improved these outcomes compared to the usual care control group.

### Effect on the primary outcome: physical fitness

This trial confirms findings from our previous single-centre study that NCRT significantly reduces physical fitness in this patient population (West et al. 2014a). Participation in the 9-week exercise programme, following completion of NCRT, had a clinically significant improvement on physical fitness levels. These improvements were evident at weeks 3 and 6 on repeat CPETs (Fig. 2). Findings may be generalizable to other surgical cancer patients with a shorter time frame between diagnosis and surgery. The increase in physical fitness may have clinical importance as the VO_2_ at AT reported at week 9 (pre-surgery) is higher than the previously established cut-off point (11.1 ml kg^−1^ min^−1^) for in-hospital post-operative morbidity (West et al. 2016). Of interest, even before initiating NCRT, the physical fitness levels of participants’ in the EMPOWER trial was lower than aged-matched healthy counterparts (Boereboom et al. 2016). Similarly, lower baseline fitness levels have also been reported in a breast cancer study (Peel et al. 2014). Future programmes should intervene with an exercise programme during the cancer staging process, as it would allow patients the opportunity to improve physical fitness levels prior to starting NCRT and maintain fitness during NCRT. Although the post-operative morbidity survey data were recorded, the exploratory nature of the outcome and small sample size preclude quantitative inference. However, currently, the WesFit trial (Identifier: NCT03509428) and the PREPARE ABC trial (Identifier: ISRCTN8223315) are currently investigating whether pre-operative exercise training improves post-operative morbidity.

### Effects on secondary outcome: health-related quality of life

The EMPOWER trial semi-structured interviews shows that NCRT adversely impacts HRQoL domains related to physical, psychosocial and behavioural functioning. Participants reported experiences of physical ill-being, social problems, behavioural/lifestyle disruptions and psychological concerns. Those on the exercise programme experienced positive changes in perceptions of physical well-being and psychological well-being. To our knowledge, no other study has explored this through semi-structured interviews apart from our preliminary work in the same population (Burke et al. 2013), where we showed that participants experiened improved vitality, a positive attitude, enhanced social connections and a sense of purpose in life a 6-week pre-operative exercise training (Burke et al. 2013). Additionally, the EMPOWER trial also shows that the 9-week exercise prehabilitation programme had a significant improvement on participants’ health status and usual activities. These positive HRQoL findings at week 9 may be of importance as psychological variables are associated with early surgical recovery (Mavros et al. 2011). To our knowledge, no studies have investigated the effects of exercise prehabilitation on HRQoL and surgical outcome. However, this area is emerging with the development of prehabiliation programmes which aim to optimise physical fitness, nutritional and psychological outcomes pre-operatively.

### Effect on the exploratory outcome: physical activity

The EMPOWER trial shows no significant changes in physical activity following NCRT or the exercise prehabilitation programme. At present, little is known about objective physical activity levels in this regard. To our knowledge, our previous pilot study was the first to report this in a non-randomised controlled trial. We showed that NCRT significantly reduced daily step count however participating in a 6-week exercise programme immediately following NCRT significantly improved physical activity outcome measures for sleep efficiency and duration. Contributing factors to the difference in these findings from our pilot study and the EMPOWER trial may be due to the difference in pre-NCRT daily step count (higher in the EMPOWER trial), study design (RCT vs. a single centre non-randomised contemporaneous controlled study) and comorbidity (50% of participants in the previous pilot study had comorbid disease compared to 8% in the EMPOWER trial). The physical activity data in EMPOWER does suggest however that the positive findings presented for physical fitness are attributable to the structured exercise training programme and not due to any significant changes in physical activity over the 9-week period. Interestingly, in both our initial pilot work and EMPOWER, we reported a low metabolic equivalent threshold (MET) scores across the cancer care pathway (from diagnosis to surgery), suggestive of light intensity activity. This may be clinically important: a MET score of 27 MET-hours per week in men with colorectal cancer is associated with a 50% reduced risk of colorectal cancer-specific mortality and overall mortality, compared with engaging in < 3 MET-hours/week (Meyerhardt et al. 2006). The MET score at week 0 and week 9 in both groups equates to between 9.1 and 9.8 MET-hours per week which is almost 60% less than that reported for disease-free survival benefits. Perhaps there is a role for issuing physical activity monitors at outpatient clinic at the point of cancer diagnosis and educate patients on the importance of moderate intensity physical activity.

### Strengths and weaknesses of this study

Strengths of this multi-centre RCT include a homogeneous group including MRI defined circumferential margin threatened (locally advanced) rectal cancer patients, the novel in-hospital programme with a clearly defined exercise intervention, prescribed individually, with training intensities derived and reported by two assessors. CPET used a constant protocol and software; analysis was by two independent physiological assessors blind to group allocation, all outcome data and independent of the intervention; and the multi-disciplinary team caring for the patients were not provided with any information regarding predictive measures (CPET variables). Additionally, the inclusion of the HRQoL which was collected at the same time points as CPET. Physical activity was measured in an objective manner using validated SenseWear activity monitors; participants in the exercise group did not wear the physical activity monitors during exercise sessions, allowing for accurate comparisons between the groups. Randomization was computerized. Data were handled using a double-entry data system. The statisticians conducting the analyses were blind to the group allocation until after the analysis was complete.

Weaknesses are that the target sample size was not achieved. This is mainly due to a change in treatment pathways at recruiting sites during the trial conduct: a lower number of patients undergoing long-course NCRT and surgery than originally forecasted became apparent (note: some NHS trusts have a variation of NCRT options for MRI threatened rectal cancer, i.e. short course NCRT with a short waiting time to surgery or neoadjuvant systemic chemotherapy prior to NCRT with a variable wait time between the end of NCRT and surgery). To overcome this challenge, the recruitment period was extended for an additional year (from December 2015 to December 2016) and three additional sites were invited to participate from 2014 to 2015 (recruitment initially started in University Hospital Southampton and University Hospital Aintree in 2013/2014). Therefore, the nature of this underpowered trial increases the false negative rate. Future work may benefit from involving an implementation scientist as part of the co-design of prehabilitation studies. Potential weaknesses also lie in the high dropout rate, the heterogeneity of the NCRT regimen (due to a change in clinical treatment pathway during the study period in University Hospital Southampton). Additionally, the recruitment uptake rate was 48%. Although there is limited literature published in the neoadjuvant setting to make comparisons against, one previous study in the same study setting reported a recruitment uptake rate of 56% (Morielli et al. 2016b). Additionally, the high risk of performance and detection bias as both participants and personnel were not blinded, and the sample population largely consisted of males. Data reporting nutrition, sarcopenia, anaemia and preoperative inflammation outcomes were not recorded as part of this study, all of which would have been added to the study.

### Conclusions

NCRT significantly reduced physical fitness levels and HRQoL. A 9-week exercise prehabilitation programme initiated following NCRT resulted in a clinical and significant increase in VO_2_ at AT and had a positive effect on HRQoL compared to a usual care control group. The findings from the EMPOWER trial informed the WesFit trial that is currently investigating whether fitness, behaviour change support and emotional support programmes delivered in the community can boost recovery rates after major cancer surgery (in collaboration with Wessex Cancer Alliance). As hospital-based programmes have been impacted during the COVID-19 pandemic, such community programmes are important to investigate.

## Supplementary Information

**Additional file 1: Supplementary Appendix 1.** The Consensus on Exercise Reporting Template (CERT). **Supplementary Appendix 2 Table S1.** Summary of CPET variables at week 0, 3, 6 and 9. **Supplementary Appendix 3.** Individual Graphical Plots. **Figure S1.** Changes in oxygen uptake at anaerobic threshold (ml.kg^-1^.min^-1^) at week 0/post-neoadjuvant cancer treatment, week 3, 6 and 9 in the exercise group. **Figure S2**. Changes in oxygen uptake at anaerobic threshold (ml.kg^-1^.min^-1^) at week 0/post-neoadjuvant cancer treatment, week 3, 6 and 9 in the usual care control group. **Supplementary Appendix 4 Table S2**. Summary of CPET variables between pre- and post-NCRT. **Supplementary Appendix 5 Table S3.** Themes, subthemes, and representative quotes from patients following neoadjuvant cancer treatment and the exercise training programme. **Supplementary Appendix 6 Table S4.** Post-Operative Morbidity Scores. **Supplementary Appendix 7 Table S5**. Summary of response to treatment and histopathology

## Data Availability

The datasets used and/or analysed during the current study are available from the corresponding author on reasonable request.

## Abbreviations

AT: Anaerobic threshold
CPET: Cardiopulmonary exercise testing
EORTC QLQ-C30: European Organization for Research and Treatment of Cancer Quality of Life Questionnaire
EQ-5D-5L: 5-level EQ-5D questionnaire
HRQoL: Health related quality of life
MET: Metabolic equivalent threshold
MRI: Magnetic resonance imaging
NCRT: Neoadjuvant chemoradiotherapy
NHS: National Health Service
POETTS: Perioperative exercise testing and training society
RECIST: Response evaluation criteria in solid tumours
TNM: Tumour, node, metastasis
VO_2_: Oxygen uptake
ymrTRG: MRI-based T-staging tumour regression grade
ypTRG: Histopathological tumour regression grading

## References

[CR1] Aaronson NK, Ahmedzai S, Bergman B, Bullinger M, Cull A, Duez NJ, Filiberti A, Flechtner H, Fleishman SB, Haes JCJM, Kaasa S, Klee M, Osoba D, Razavi D, Rofe PB, Schraub S, Sneeuw K, Sullivan M, Takeda F (1993). The European Organization for Research and Treatment of Cancer QLQ-C30: a quality-of-life instrument for use in international clinical trials in oncology. J Natl Cancer Inst.

[CR2] Belle V (2002). Statistical rules of thumb. Chichester.

[CR3] Boereboom CL, Phillips BE, Williams JP, Lund JN (2016). A 31-day time to surgery compliant exercise training programme improves aerobic health in the elderly. Tech Coloproctol.

[CR4] Burke SM, Brunet J, Sabiston CM, Jack S, Grocott MP, West MA (2013). Patients’ perceptions of quality of life during active treatment for locally advanced rectal cancer: the importance of preoperative exercise. Support Care Cancer.

[CR5] Carlisle J, Swart M (2007). Mid-term survival after abdominal aortic aneurysm surgery predicted by cardiopulmonary exercise testing. Br J Surg.

[CR6] Du D, Su Z, Wang D, Liu W, Wei Z (2017). Optimal interval to surgery after neoadjuvant chemoradiotherapy in rectal cancer: a systematic review and meta-analysis. Clin Colorectal Cancer.

[CR7] Dworak O, Keilholz L, Hoffmann A (1997). Pathological features of rectal cancer after preoperative radiochemotherapy. Int J Color Dis.

[CR8] Fanke AJ, Parekh H, Starr JS, Tan SA, Iqbal A, George TJ (2018). Total neaodjuvant therapy: a shifting paradigm in locally advanced rectal cancer management. Clin Colorectal Cancer.

[CR9] Glimelius B, Pahlman L, Cervantes A, ESMO Guidelines Working Group. Rectal cancer: ESMO Clinical Practice Guidelines for diagnosis, treatment and follow-up. Ann Oncol. 2010. 10.1093/annonc/mdq170.

[CR10] Grocott MP, Browne JP, Van der Meulen J, Matejowsky C, Mutch M, Hamilton MA (2007). The postoperative morbidity survey was validated and used to describe morbidity after major surgery. J Clin Epidemiol.

[CR11] Habr-Gama A, Perez RO, Sabbaga J, Nadalin W, São Julião GP, Gama-Rodrigues J (2009). Increasing the rates of complete response to neoadjuvant chemoradiotherapy for distal rectal cancer: results of a prospective study using additional chemotherapy during the resting period. Dis Colon Rectum.

[CR12] Heldens AFJM, Bongers BC, de Vos-Geelen J, van Meeteren NLU, Lenssen AF (2016). Feasibility and preliminary effectiveness of a physical exercise training program during neoadjuvant chemoradiotherapy in individual patients with rectal cancer prior to major elective surgery. Eur J Surg Oncol.

[CR13] Kothmann E, Batterham AM, Owen SJ, Turley AJ, Cheesman M, Parry A, Danjoux G (2009). Effect of short-term exercise training on aerobic fitness in patients with abdominal aortic aneurysms: a pilot study. Br J Anaesth.

[CR14] Lee CHA, Kong JC, Ismail H, Riedel B, Heriot A (2018). Systematic review and meta-analysis of objective assessment of physical fitness in patients undergoing colorectal cancer surgery. Dis Colon Rectum.

[CR15] Levett DZH, Jack S, Swart M, Carlisle J, Wilson J, Snowden C, Riley M, Danjoux G, Ward SA, Older P, Grocott MPW, Perioperative Exercise Testing and Training Society (POETTS) (2018). Perioperative cardiopulmonary exercise testing (CPET): consensus clinical guidelines on indications, organisation, conduct, and physiological interpretation. Br J Anaesth.

[CR16] Loughney L, West MA, Kemp GJ, Rossiter HB, Burke SM, Cox T, Barben CP, Mythen MG, Calverley P, Palmer DH, Grocott MPW, Jack S (2016). The effects of neoadjuvant chemoradiotherapy and an in-hospital exercise training programme on physical fitness and quality of life in locally advanced rectal cancer patients (The EMPOWER Trial): study protocol for a randomised controlled trial. Trials..

[CR17] Loughney LA, West MA, Kemp GJ, Grocott MPW, Jack S. Exercise training interventions for people with cancer during cancer treatment before or after surgery. Cochrane Database Syst Rev. 2018. 10.1002/14651858.CD012280. 10.1002/14651858.CD012280.pub2 PMC6517034 30536366

[CR18] Mavros MN, Athanasiou S, Gkegkes ID, Polyzos KA, Peppas G, Falagas ME (2011). Do psychological variables affect early surgical recovery?. PLoS One.

[CR19] Meyerhardt JA, Giovannucci EL, Holmes MD, Chan AT, Chan JA, Colditz GA, Fuchs CS (2006). Physical activity and survival after colorectal cancer diagnosis. J Clin Oncol.

[CR20] Moran J, Wilson F, Guinan E, McCormick P, Hussey J, Moriarty J (2016). Role of cardiopulmonary exercise testing as a risk-assessment method in patients undergoing intra-abdominal surgery: a systematic review. Br J Anaesth.

[CR21] Morielli AR, Boule NG, Usmani N, Joseph K, Tankel K, Severin D (2018). Predictors of adherence to aerobic exercise in rectal cancer patients during and after neoadjuvant chemoradiotherapy. Psychol Health Med.

[CR22] Morielli AR, Usmani N, Boule NG, Severin D, Tankel K, Nijjar T (2016). Exercise motivation in rectal cancer patients during and after neoadjuvant chemoradiotherapy. Support Care Cancer.

[CR23] Morielli AR, Usmani N, Boule NG, Severin D, Tankel K, Nijjar (2018). Exercise during and after neoadjuvant rectal cancer treatment (the EXERT trial): study protocol for a randomised controlled trial. Trials.

[CR24] Morielli AR, Usmani N, Boule NG, Tankel K, Severin, Nijjar (2016). A phase I study examining the feasibility and safety of an aerobic exercise intervention in patients with rectal cancer during and after neoadjuvant chemoradiotherapy. Oncol Nurs Forum.

[CR25] Moug SJ, Mutrie N, Barry SJE, Mackay G, Steele RJC, Boachie C, Buchan C, Anderson AS (2019). Prehabilitation is feasible in patients with rectal cancer undergoing neoadjuvant chemoradiotherapy and may minimize physical deterioration: results from the Rex trial. Color Dis.

[CR26] NICE: National Institute for Health and Care Excellence: Colorectal cancer (update): preoperative radiotherapy ad chemoradiotherapy for rectal cancer. NICE guideline NG15 last updated January 2020. 32729997

[CR27] Nilsson PJ, van Etten B, Hospers GA, Pahlman L, van de Velde CJH, Beets-Tan RGH (2013). Short-course radiotherapy followed by neo-adjuvant chemotherapy in locally advanced rectal cancer--the RAPIDO trial. BMC Cancer.

[CR28] Patel UB, Blomqvist LK, Taylor F, George C, Guthrie A, Bees N, Brown G (2012). MRI after treatment of locally advanced rectal cancer: how to report tumor response: the MERCURY experience. AJR..

[CR29] Patel UB, Taylor F, Blomqvist L, George C, Evans H, Tekkis P, Quirke P, Sebag-Montefiore D, Moran B, Heald R, Guthrie A, Bees N, Swift I, Pennert K, Brown G (2011). Magnetic resonance imaging-detected tumor response for locally advanced rectal cancer predicts survival outcomes: MERCURY experience. J Clin Oncol.

[CR30] Pearse RM, Moreno RP, Bauer P, Pelosi P, Metnitz P, Spies C, Vallet B, Vincent JL, Hoeft A, Rhodes A, European Surgical Outcomes Study (EuSOS) group for the Trials groups of the European Society of Intensive Care Medicine and the European Society of Anaesthesiology (2012). Mortality after surgery in Europe: a 7 day cohort study. Lancet..

[CR31] Peel AB, Thomas SM, Dittus K, Jones LW, Lakoski SG (2014). Cardiorespiratory fitness in breast cancer patients: a call for normative values. J Am Heart Assoc.

[CR32] Pucciarelli S, Gagliardi G, Maretto I, Lonardi S, Friso ML, Urso E (2009). Long-term oncologic results and complications after preoperative chemoradiotherapy for rectal cancer: a single-institution experience after a median follow-up of 95 months. Ann Surg Oncol.

[CR33] Rabin R, de Charro F (2001). EQ-5D: a measure of health status from the EuroQol Group. Ann Med.

[CR34] Singh F, Galvao DA, Newton RU, Spry NA, Baker MK, Taaffe DR (2018). Feasibility and preliminary efficacy of a 10-week resistance and aerobic exercise intervention during neoadjuvant chemoradiation treatment in rectal cancer patients. Inter Cancer Ther.

[CR35] Slade SC, Dionne CE, Underwood M, Buchbinder R (2016). Consensus on Exercise Reporting Template (CERT): explanation and elaboration statement. Br J Sports Med.

[CR36] Snowden CP, Prentis JM, Anderson HL, Roberts DR, Randles D, Renton M, Manas DM (2010). Submaximal cardiopulmonary exercise testing predicts complications and hospital length of stay in patients undergoing major elective surgery. Ann Surg.

[CR37] West MA, Asher R, Browning M, Minto G, Swart M, Richardson (2016). Validation of preoperative cardiopulmonary exercise testing-derived variables to predict in-hospital morbidity after major colorectal surgery. Br J Surg.

[CR38] West MA, Loughney L, Barben CP, Sripadam R, Kemp GJ, Grocott MP (2014). The effects of neoadjuvant chemoradiotherapy on physical fitness and morbidity in rectal cancer surgery patients. Eur J Surg Oncol.

[CR39] West MA, Loughney L, Lythgoe D, Barben CP, Sripadam R, Kemp GJ (2014). Effect of prehabilitation on objectively measured physical fitness after neoadjuvant treatment in preoperative rectal cancer patients: a blinded interventional pilot study. Br J Anaesth.

[CR40] Wijeysundera DN, Pearse RM, Shulman MA, Abbott TEF, Torres E, Ambosta A (2018). Assessment of functional capacity before major non-cardiac surgery: an international, prospective cohort study. Lancet..

[CR41] Wilson RJ, Davies S, Yates D, Redman J, Stone M (2010). Impaired functional capacity is associated with all-cause mortality after major elective intra-abdominal surgery. Br J Anaesth.

[CR42] Wyrwicz L, Temnyk M, Spalek M. The addition of oxaliplatin increases pathological complete response: a meta-analysis of randomized controlled trials on radiochemotherapy in rectal cancer. Ann Oncol. 2016. 10.1093/annonc/mdw199.217.

