# Correction: The effects of neoadjuvant chemoradiotherapy and an in-hospital exercise training programme on physical fitness and quality of life in locally advanced rectal cancer patients: a randomised controlled trial (The EMPOWER Trial)

**DOI:** 10.1186/s13741-025-00611-y

**Published:** 2025-12-04

**Authors:** Lisa Loughney, Malcolm A. West, Helen Moyses, Andrew Bates, Graham J. Kemp, Lesley Hawkins, Judit Varkonyi-Sepp, Shaunna Burke, Christopher P. Barben, Peter M. Calverley, Trevor Cox, Daniel H. Palmer, Michael G. Mythen, Michael P. W. Grocott, Sandy Jack

**Affiliations:** 1 Anaesthesia and Critical Care Research Area, NIHR Biomedical Research Centre, University Hospital Southampton NHS Foundation Trust, Road, Southampton, UK; 2 Integrative Physiology and Critical Illness Group, Clinical and Experimental Sciences, Faculty of Medicine, University of Southampton, Southampton, UK; 3 ExWell Medical, Dublin, Ireland; 4 Cancer Sciences Unit, Faculty of Medicine, University of Southampton, Southampton, UK; 5 Departments of Anaesthesia and Critical Care, Royal Bournemouth NHS Foundation Trust, Bournemouth, UK; 6 Department of Musculoskeletal Biology and MRC – Arthritis Research UK Centre for Integrated research into Musculoskeletal Ageing (CIMA), Faculty of Health and Life Sciences, University of Liverpool, Liverpool, UK; 7 Faculty of Biological Sciences, School of Biomedical Sciences, University of Leeds, Leeds, UK; 8 Department of Colorectal Surgery, Aintree University Hospitals NHS Foundation Trust, Liverpool, UK; 9 Institute of Ageing and Chronic Disease, University of Liverpool, Liverpool, UK; 10 Cancer Research UK Liverpool Cancer Trials Unit, University of Liverpool, Liverpool, UK; 11 Institute of Translational Medicine, University of Liverpool, Liverpool, UK; 12 Anaesthesia and Critical Care, University College London, London, UK

**Correction: Perioper Med 10, 23 (2021)**

**https://doi.org/10.1186/s13741-021-00190-8**

Following the publication of the original article (Loughney et al. 2021), the authors identified an error in the Competing interests section. The section should read:

**Competing interests**

Mike Grocott is the Editor-in-Chief of the Perioperative Medicine.

**The old statement of the Competing interests**: The authors declare that they have no competing interests.

The Competing interests section has been updated above and the original article (Loughney et al. 2021) has been corrected.
