# Supplementary material for: The effects of neoadjuvant chemoradiotherapy and an in-hospital exercise training programme on physical fitness and quality of life in locally advanced rectal cancer patients: a randomised controlled trial (The EMPOWER Trial)
Source: Perioper Med (Lond). 2021 Jun 22;10:23. doi: 10.1186/s13741-021-00190-8 (PMC8216760; doi:10.1186/s13741-021-00190-8)
Supplement: Supplementary file 1 — Additional file 1: Supplementary Appendix 1. The Consensus on Exercise Reporting Template (CERT). Supplementary Appendix 2 Table S1. Summary of CPET variables at week 0, 3, 6 and 9. Supplementary Appendix 3. Individual Graphical Plots. Figure S1. Changes in oxygen uptake at anaerobic threshold (ml.kg-1.min-1) at week 0/post-neoadjuvant cancer treatment, week 3, 6 and 9 in the exercise group. Figure S2. Changes in oxygen uptake at anaerobic threshold (ml.kg-1.min-1) at week 0/post-neoadjuvant cancer treatment, week 3, 6 and 9 in the usual care control group. Supplementary Appendix 4 Table S2. Summary of CPET variables between pre- and post-NCRT. Supplementary Appendix 5 Table S3. Themes, subthemes, and representative quotes from patients following neoadjuvant cancer treatment and the exercise training programme. Supplementary Appendix 6 Table S4. Post-Operative Morbidity Scores. Supplementary Appendix 7 Table S5. Summary of response to treatment and histopathology [file 13741_2021_190_MOESM1_ESM.docx]

**Supplementary Appendix 1. The Consensus on Exercise Reporting Template (CERT)**

1. ***Type of exercise equipment used:***

An electromagnetically braked cycle ergometer (Optibike Ergoline GmbH^TM^, Germany). The exercise training consisted of 40 min (including 5 min warm-up and 5 min cool-down) of interval training. The programme was preloaded on a chip-and-pin card which executed the interval intensities automatically.

1. ***Qualifications, teaching/supervising expertise, and/or training undertaken by the exercise instructor:***

The exercise intervention was supervised by hospital staff with a minimal qualification of Basic Life Support. All staff were trained to recognise abnormal haemodynamic response to exercise and to initiate appropriate response.^1^

Professional background varied between hospital sites, either physiotherapy assistants, research exercise physiologists or research nurses, trained by the bicycle ergometer suppliers. All exercise instructors were trained in Good Clinical Practice and delegated authority by local Principal Investigators.

1. ***Individual or group exercise:***

Wherever possible, dependent on availability, trial participants exercised in pairs, to provide camaraderie.

1. ***Exercise supervision and delivery:***

The exercise sessions were supervised and delivered in hospital. The supervisors ensured continuing desire for trial participation and questioned the participant about any medical consultations, subsequent to previous exercise visits. In addition, basic observations were taken for heart rate, blood pressure and oxygen saturations. The participants were reminded to inform the supervisor in the case of chest pain, sudden onset shortness of breath and dizziness.^1^

1. ***How was fidelity/ adherence to exercise measured and reported:***

The exercise session completion rates and intensities were recorded on a pin and chip card which were inserted into the training ergometer. This chip and pin system recorded live patient data (heart rate and cadence) during the sessions, which was downloaded to provide post hoc analysis of adherence. Patient adherence was reported as a percentage of prescribed exercise sessions that were attended.

1. ***Description of motivation strategies:***

All exercise sessions were supervised throughout as described above. No specific motivational strategies were adopted, although presence of the supervisor and pairing of trial participants was reported to increase motivation and adherence by our patient ambassadors.

1. ***Exercise progression:***

Exercise intensity was prescribed onto a chip and pin card, using the participant’s physiological variables determined by week 0 Cardiopulmonary Exercise Testing (CPET). Participants repeated CPET every 3 weeks and the training programme was modified accordingly, ensuring consistent, progressive and individualized intensities for all participants.

1. ***Detailed description of the structured, tailored and responsive exercise training sessions:***

Exercise training consisted of 40 min (including 5 min warm-up and 5 min cool-down) of high intensity interval aerobic training on an electromagnetically braked cycle ergometer (Optibike Ergoline GmbH^TM^, Germany). Participants randomised to the exercise group attended an in-hospital, structured, responsive exercise training programme. Three sessions per week were performed for 9 weeks. Participants wore a heart rate monitor throughout the session which reported pulse rate onto the ergometer screen.

Training intensities were derived from the individual’s physiological variables determined at week 0 CPET and loaded onto a chip and pin card. This card was inserted into the cycle ergometer and participants instructed to pedal at a cadence of 60-65 revolution per minute, according to a visible reading. Sessions lasted for 40 min, starting with a 5 min warm-up at 8 watts. After 5 min warm-up, at 8 watts, the interval components began with 3 min, moderate intensity, at a power output equivalent to 80 % of that achieved at AT. This was followed by high intensity exercise, for 2 min at a work rate equal to 50 % of the difference in work rate between AT and peak oxygen uptake, termed 50%Δ.

Algebraic calculation of training intensity:

Moderate-intensity exercise: (Work load at VO_2_ at AT - 2/3 of CPET work ramp) × 80 %

Severe-intensity exercise: ((Work load at VO_2_ at Peak - Work load at VO_2_ at AT - 2/3 of CPET work ramp) × 50 %) + Work load at VO_2_ at AT

NB - VO_2_ at AT = Oxygen uptake at Anaerobic Threshold. VO_2_ at Peak = Oxygen uptake at Peak exercise.

This 5 min interval was repeated 6 times, followed by a 5 min recovery period at 0-5 watts. In total, exercise sessions lasted for 40 mins.


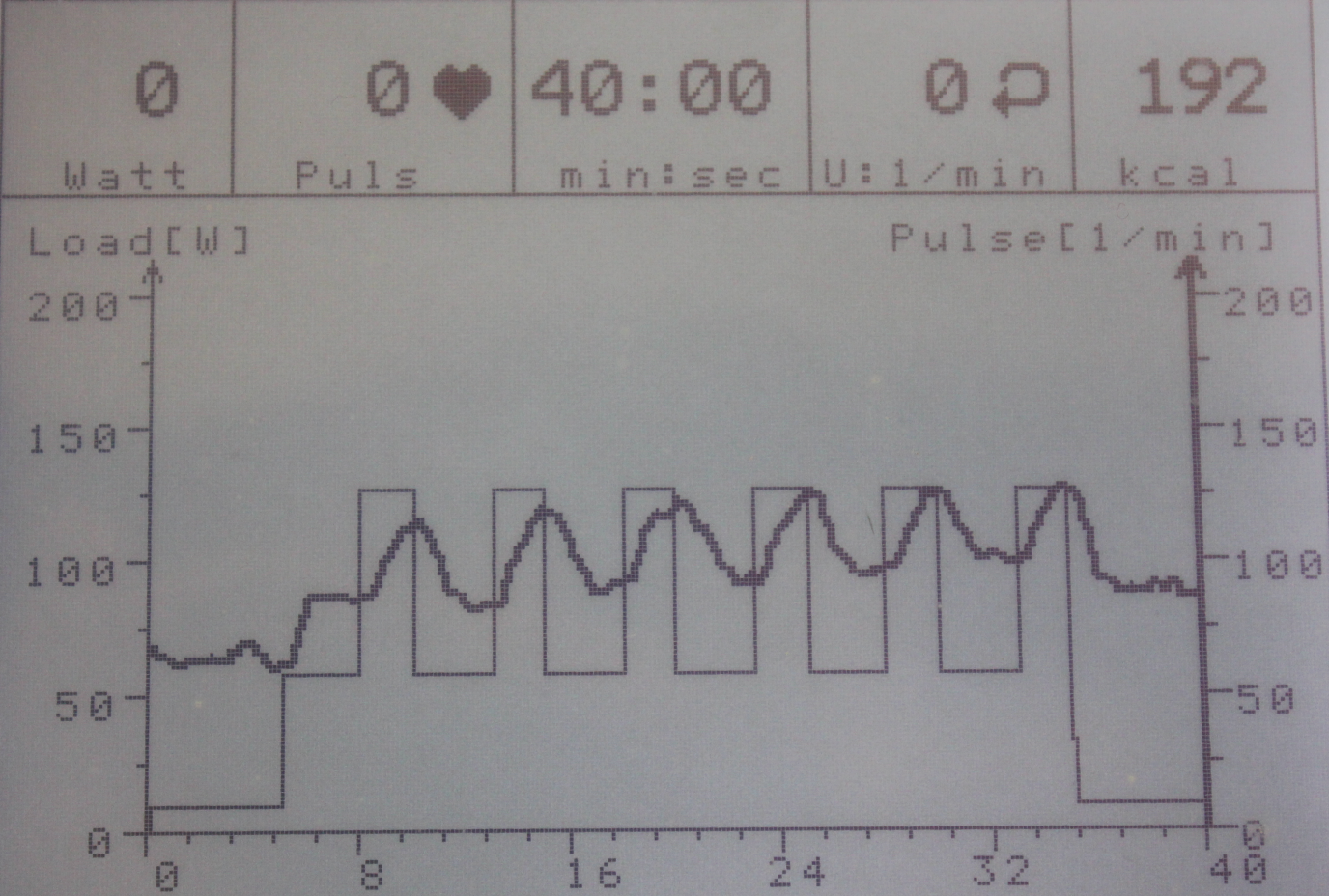


Photo of visual output from the training ergometer showing the end screen of the interval training programme. The first 5 min of exercise was a warm-up phase followed by 30 min split into 3 min (moderate intensity) and 2 min (high intensity) exercise intervals. The final 5 min was the cool-down phase. Total exercise time is 40 min.

1. ***Home programme component:***

All trial exercise participation took place in-hospital. Participants were monitored over 72 consecutive hours, for home activity levels using a multi-sensory accelerometer.

1. ***Non-exercise components:***

We did not perform non-exercise components to this trial.

1. ***Type and number of adverse events:***

Trial participants were assessed prior to commencement of exercise sessions and CPET. This consisted of questioning the patient with a blood pressure and heart rate check.

Adverse events were deemed attributable if occurring within 30 min of exercise or CPET. No serious adverse events were reported. One trial participant experienced an episode of pre-syncope following an exercise session. The participant was reviewed by the hospital medical team and General Practitioner and deemed fit to continue with the exercise intervention.

1. ***Setting in which the exercise was performed:***

The protocol stipulates that exercise sessions should be performed in-hospital. Sessions took place in the physiotherapy rehabilitation department or designated research physiology laboratory at each NHS site. This was determined according to facility available at participating sites.

1. ***Extent of adherence:***

During this trial, a total of 91% of the sessions were completed by participants, according to the prescription.

**Supplementary Appendix 2 Table 1. Summary of CPET variables at week 0, 3, 6 and 9**

| **CPET variables** | **Week** | **Exercise** | | **Control** | |
| --- | --- | --- | --- | --- | --- |
|  |  | **n** |  | **n** |  |
| VO_2_ at AT (ml.kg^-1^.min^-1^) | Week 0 | 15 | 11.6 (3.4) | 13 | 10.8 (2.5) |
|  | Week 3 | 14 | 13.7 (4.4) | 11 | 12.2 (2.2) |
|  | Week 6 | 14 | 13.6 (4.4) | 12 | 12.0 (2.0) |
|  | Week 9 | 13 | 15.0 (4.2) | 12 | 11.5 (2.5) |
|  |  |  |  |  |  |
| VO_2_ at AT (L.min^-1^) | Week 0 | 15 | 0.9 (0.4) | 13 | 0.8 (0.2) |
|  | Week 3 | 14 | 1.1 (0.5) | 11 | 0.9 (0.2) |
|  | Week 6 | 14 | 1.0 (0.4) | 12 | 0.9 (0.2) |
|  | Week 9 | 13 | 1.2 (0.5) | 12 | 0.9 (0.2) |
|  |  |  |  |  |  |
| VO_2_ at Peak (ml.kg^-1^.min^-1^) | Week 0 | 15 | 21.4 (7.7) | 13 | 19.5 (4.1) |
|  | Week 3 | 14 | 24.3 (7.0) | 11 | 21.9 (2.7) |
|  | Week 6 | 14 | 23.6 (8.7) | 12 | 21.9 (4.4) |
|  | Week 9 | 13 | 27.1 (8.7) | 12 | 22.9 (3.8) |
|  |  |  |  |  |  |
| VO_2_ at Peak (L.min^-1^) | Week 0 | 15 | 1.6 (0.7) | 13 | 1.5 (0.4) |
|  | Week 3 | 14 | 1.8 (0.7) | 11 | 1.7 (0.3) |
|  | Week 6 | 14 | 1.8 (0.7) | 12 | 1.7 (0.5) |
|  | Week 9 | 13 | 2.1 (0.9) | 12 | 1.8 (0.5) |
|  |  |  |  |  |  |
| BMI (kg.m^-2^) | Week 0 | 15 | 25.7 (3.8) | 13 | 26.2 (3.2) |
|  | Week 3 | 14 | 25.5 (3.6) | 11 | 26.3 (3.3) |
|  | Week 6 | 14 | 25.8 (3.8) | 12 | 26.3 (3.1) |
|  | Week 9 | 13 | 26.1 (3.8) | 12 | 26.2 (3.1) |
| BSA (m^2^) |  |  |  |  |  |
|  | Week 0 | 15 | 1.9 (0.2) | 13 | 1.9 (0.1) |
|  | Week 3 | 14 | 1.9 (0.2) | 11 | 1.9 (0.1) |
|  | Week 6 | 14 | 1.9 (0.2) | 12 | 1.9 (0.1) |
|  | Week 9 | 13 | 1.9 (0.2) | 12 | 1.9 (0.1) |
| Ramp (W) |  |  |  |  |  |
|  | Week 0 | 15 | 15.0 (15, 20) | 13 | 20.0 (15, 20) |
|  | Week 3 | 14 | 17.5 (15, 20) | 11 | 20.0 (15, 20) |
|  | Week 6 | 14 | 17.5 (15, 20) | 12 | 20.0 (15, 20) |
|  | Week 9 | 13 | 15.0 (15, 20) | 12 | 20.0 (15, 20) |
| Rest HR (beat.min^-1^) |  |  |  |  |  |
|  | Week 0 | 15 | 77.8 (13.0) | 13 | 80.0 (12.6) |
|  | Week 3 | 14 | 81.9 (14.5) | 11 | 83.6 (13.4) |
|  | Week 6 | 14 | 78.0 (12.6) | 12 | 76.9 (10.6) |
|  | Week 9 | 13 | 72.8 (11.6) | 12 | 75.8 (10.7) |
|  |  |  |  |  |  |
| AT HR (beat.min^-1^) | Week 0 | 15 | 99.3 (11.2) | 13 | 101.8 (12.9) |
|  | Week 3 | 14 | 102.7 (11.9) | 11 | 108.7 (11.8) |
|  | Week 6 | 14 | 101.4 (13.5) | 12 | 99.3 (10.9) |
|  | Week 9 | 13 | 98.4 (11.1) | 12 | 95.7 (12.7) |
|  |  |  |  |  |  |
| Peak HR (beat.min^-1^) | Week 0 | 15 | 135.4 (14.6) | 13 | 143.7 (17.3) |
|  | Week 3 | 14 | 141.5 (17.2) | 11 | 151.7 (19.4) |
|  | Week 6 | 14 | 136.9 (19.7) | 12 | 144.1 (21.9) |
|  | Week 9 | 13 | 140.2 (18.2) | 12 | 148.7 (18.9) |
| WR at AT (W) |  |  |  |  |  |
|  | Week 0 | 15 | 61.9 (37.8) | 13 | 62.4 (26.0) |
|  | Week 3 | 14 | 76.2 (45.9) | 11 | 71.1 (16.5) |
|  | Week 6 | 14 | 79.1 (45.2) | 12 | 64.1 (14.3) |
|  | Week 9 | 13 | 85.3 (40.7) | 12 | 64.0 (15.1) |
|  |  |  |  |  |  |
| WR at Peak (W) | Week 0 | 15 | 141.4 (59.8) | 13 | 142.1 (42.2) |
|  | Week 3 | 14 | 159.3 (61.9) | 11 | 151.0 (37.4) |
|  | Week 6 | 14 | 162.5 (68.4) | 12 | 149.2 (47.2) |
|  | Week 9 | 13 | 178.2 (74.1) | 12 | 160.7 (39.5) |
|  |  |  |  |  |  |
| V_E_/VCO_2_ at AT | Week 0 | 15 | 31.8 (4.8) | 13 | 33.2 (6.6) |
|  | Week 3 | 14 | 31.5 (3.8) | 11 | 30.3 (2.2) |
|  | Week 6 | 14 | 32.5 (3.7) | 12 | 30.3 (2.0) |
|  | Week 9 | 13 | 31.4 (3.6) | 12 | 30.3 (2.6) |
|  |  |  |  |  |  |
| V_E_/VCO_2_ at Peak | Week 0 | 15 | 34.8 (6.6) | 13 | 35.9 (8.3) |
|  | Week 3 | 14 | 34.6 (3.7) | 11 | 33.9 (4.1) |
|  | Week 6 | 14 | 34.1 (3.6) | 12 | 32.7 (2.3) |
|  | Week 9 | 13 | 35.3 (2.9) | 12 | 33.0 (3.5) |
| V_E_/VO_2_ at AT |  |  |  |  |  |
|  | Week 0 | 15 | 27.6 (4.4) | 13 | 28.2 (5.7) |
|  | Week 3 | 14 | 28.1 (3.8) | 11 | 27.3 (2.8) |
|  | Week 6 | 14 | 28.3 (4.1) | 12 | 27.0 (2.9) |
|  | Week 9 | 13 | 26.8 (2.6) | 12 | 26.0 (3.2) |
|  |  |  |  |  |  |
| V_E_/VO_2_ at Peak | Week 0 | 15 | 42.1 (8.1) | 13 | 42.7 (9.4) |
|  | Week 3 | 14 | 42.6 (5.2) | 11 | 41.8 (6.1) |
|  | Week 6 | 14 | 40.4 (5.5) | 12 | 40.3 (4.1) |
|  | Week 9 | 13 | 41.2 (4.6) | 12 | 40.0 (5.1) |
|  |  |  |  |  |  |
| PETCO_2_ at AT (mm Hg) | Week 0 | 15 | 39.0 (4.6) | 13 | 36.3 (6.0) |
|  | Week 3 | 14 | 38.8 (2.8) | 11 | 38.9 (3.4) |
|  | Week 6 | 14 | 38.7 (2.6) | 12 | 39.2 (2.8) |
|  | Week 9 | 13 | 38.7 (2.6) | 12 | 39.1 (3.4) |
|  |  |  |  |  |  |
| PETCO_2_ at Peak (mm Hg) | Week 0 | 15 | 9.0 (3.6) | 13 | 8.3 (2.3) |
|  | Week 3 | 14 | 10.2 (4.2) | 11 | 8.9 (2.1) |
|  | Week 6 | 14 | 10.1 (3.3) | 12 | 9.3 (1.5) |
|  | Week 9 | 13 | 11.8 (4.3) | 12 | 9.7 (2.4) |
|  |  |  |  |  |  |
| PETCO_2_ at Peak (mm Hg) | Week 0 | 15 | 35.7 (4.8) | 13 | 34.1 (7.1) |
|  | Week 3 | 14 | 35.0 (3.5) | 11 | 35.0 (4.2) |
|  | Week 6 | 14 | 35.6 (3.0) | 12 | 36.2 (2.7) |
|  | Week 9 | 13 | 34.5 (2.3) | 12 | 36.2 (3.8) |
|  |  |  |  |  |  |
| VO_2_/HR at AT (ml.beat^-1^) | Week 0 | 15 | 9.0 (3.6) | 13 | 8.3 (2.3) |
|  | Week 3 | 14 | 10.2 (4.2) | 11 | 8.9 (2.1) |
|  | Week 6 | 14 | 10.1 (3.3) | 12 | 9.3 (1.5) |
|  | Week 9 | 13 | 11.8 (4.3) | 12 | 9.7 (2.4) |
| VO_2_/HR at Peak (ml.beat^-1^) |  |  |  |  |  |
|  | Week 0 | 15 | 11.8 (4.5) | 13 | 10.7 (2.7) |
|  | Week 3 | 14 | 12.9 (4.1) | 11 | 11.4 (2.4) |
|  | Week 6 | 14 | 12.6 (3.8) | 12 | 11.8 (2.0) |
|  | Week 9 | 13 | 14.8 (5.1) | 12 | 12.5 (3.1) |
| Breathing reserve (l.min) |  |  |  |  |  |
|  | Week 0 | 15 | 41.8 (23.6) | 13 | 50.0 (22.0) |
|  | Week 3 | 14 | 34.0 (18.0) | 11 | 40.5 (16.1) |
|  | Week 6 | 14 | 38.0 (24.6) | 12 | 42.4 (18.0) |
|  | Week 9 | 13 | 24.8 (21.0) | 12 | 41.6 (14.5) |

Data are mean (SD) or median (IQR).

Abbreviations: VO_2_ at AT (oxygen uptake at anaerobic threshold), VO_2_ at Peak (oxygen uptake at peak exercise), BMI (body mass index); BSA (body surface area); WR at AT (work rate at anaerobic threshold), WR at Peak (work rate at peak exercise), V_E_/VCO_2_ at AT (ventilatory equivalent for carbon dioxide at anaerobic threshold), V_E_/VCO_2_ at Peak (ventilatory equivalent for carbon dioxide at peak exercise), V_E_/VO_2_ at AT (ventilatory equivalent for oxygen at anaerobic threshold), V_E_/VO_2_ at Peak (ventilatory equivalent for oxygen at peak exercise), PETCO_2_ at AT (end-tidal carbon dioxide at anaerobic threshold), PETCO_2_ at Peak (end-tidal carbon dioxide at peak exercise), VO_2_/HR at AT (oxygen pulse at anaerobic threshold), VO_2_/HR at Peak (oxygen pulse at peak exercise).

**Supplementary Appendix 3. Individual Graphical Plots**

**Figure 1.** Changes in oxygen uptake at anaerobic threshold (ml.kg^-1^.min^-1^) at week 0/post-neoadjuvant cancer treatment, week 3, 6 and 9 in the exercise group.

**Figure 2.** Changes in oxygen uptake at anaerobic threshold (ml.kg^-1^.min^-1^) at week 0/post-neoadjuvant cancer treatment, week 3, 6 and 9 in the usual care control group.

**Supplementary Appendix 4 Table 2. Summary of CPET variables between pre- and post-NCRT**

| **CPET Variables** | **Pre-NCRT (n=28)** | **Post-NCRT (n=28)** |
| --- | --- | --- |
| VO_2_ at AT (ml.kg^-1^.min^-1^) | 12.6 (3.3) | 11.2 (3.0) |
| VO_2_ at AT (ml.min^-1^) | 1.0 (0.3) | 0.9 (0.3) |
| VO_2_ at Peak (ml.kg^-1^.min^-1^) | 22.2 (7.8) | 20.5 (6.2) |
| VO_2_ at Peak (ml.min^-1^) | 1.7 (0.6) | 1.6 (0.6) |
| BMI (kg.m^-2^) | 26.4 (3.6) | 25.9 (3.5) |
| BSA (m^2^) | 1.9 (0.2) | 1.9 (0.2) |
| Ramp (W) | 19.3 (5.9) | 18.2 (6.3) |
| Rest HR (beat.min^-1^) | 76.8 (19.9) | 78.8 (12.6) |
| AT HR (beat.min^-1^) | 103.6 (14.3) | 100.5 (11.9) |
| Peak HR (beat.min^-1^) | 141.6 (18.5) | 139.3 (16.1) |
| WR at AT (W) | 73.2 (39.9) | 62.1 (32.2) |
| WR at Peak WR | 153.6 (57.1) | 141.8 (51.5) |
| V_E_/VCO_2_ at AT | 30.8 (4.3) | 32.5 (5.6) |
| V_E_/VCO_2_ at Peak | 39.4 (6.9) | 35.3 (7.3) |
| V_E_/VO_2_ at AT | 26.5 (3.9) | 27.9 (4.9) |
| V_E_/VO_2_ at Peak | 33.5 (5.3) | 42.4 (8.6) |
| PETCO_2_ at AT (mm Hg) | 38.8 (4.1) | 37.8 (5.4) |
| PETCO_2_ at Peak (mm Hg) | 35.9 (4.5) | 34.9 (5.9) |
| VO_2_/HR at AT (ml.beat^-1^) | 9.5 (2.8) | 8.7 (3.0) |
| VO_2_/HR at Peak (ml.beat^-1^) | 12.1 (3.8) | 11.3 (3.8) |
| Breathing reserve (l.min) | 44.8 (21.3) | 45.5 (22.8) |

Data are presented as mean (SD). Abbreviations: neoadjuvant chemoradiotherapy (NCRT); VO_2_ at AT (oxygen uptake at anaerobic threshold), VO_2_ at Peak (oxygen uptake at peak exercise), BMI (body mass index); BSA (body surface area); WR at AT (work rate at anaerobic threshold), WR at Peak (work rate at peak exercise), V_E_/VCO_2_ at AT (ventilatory equivalent for carbon dioxide at anaerobic threshold), V_E_/VCO_2_ at Peak (ventilatory equivalent for carbon dioxide at peak exercise), V_E_/VO_2_ at AT (ventilatory equivalent for oxygen at anaerobic threshold), V_E_/VO_2_ at Peak (ventilatory equivalent for oxygen at peak exercise), PETCO_2_ at AT (end-tidal carbon dioxide at the anaerobic threshold), PETCO_2_ at Peak (end-tidal carbon dioxide at peak exercise), VO_2_/HR at AT (oxygen pulse at anaerobic threshold), VO_2_/HR at Peak (oxygen pulse at peak exercise)

**Supplementary Appendix 5 Table 3. Themes, subthemes, and representative quotes from patients following neoadjuvant cancer treatment and the exercise training programme**

| **Main theme** | **Subtheme** | **Example quote** |
| --- | --- | --- |
| **Effects of treatment on HRQoL (n=28)** | | |
| **Physical ill-being** | *Persistent fatigue* | *“...it drains you physically. You sit down in the armchair, and the next thing you know you are fast asleep. That has gone on for five weeks, so that [falling asleep] has really changed my whole outlook on my life, because I am so used to getting out and doing things, and walking and going into town and having a look around. But I have not been able to do that, because I have been so tired. So the treatment has kind of restricted me to what I can do. I try to do as much as I can, but I get so far and then I have got to stop, because I am so fatigued.”* |
|  | *Pain and discomfort* | *“I think of my grandkids and I don’t want to die. It’s the last thing but then I couldn’t have cared less as I was in that much pain. I’ve never felt like that in my life before”* |
|  | *Impaired bodily functions* | *“I was going to the bathroom every 25-30 minutes, I didn’t get any sleep. I recently started sleeping on my right hand side and my left hand side again, but last night I couldn’t. If I slept on my right hand side or left hand side, I had the runs, it just disrupted the whole system.”* |
|  | *Reduced physical fitness* | *“I’m not as fit as I was at the beginning, when I was first diagnosed...I was biking to and from work every day. Since the treatment started, I haven’t been able to really do that.”* |
| **Social disruptions** | *Social isolation* | *“I don’t see me mates at work, I don’t see me mates at all...I haven’t been anywhere, just sitting in the house.”* |
|  | *Changed social interactions* | *“I’ve got a young grandchild and some younger family members and normally I’m very robust with them and quite active with them. And that hasn’t happened. I haven’t been as active as I want to be with them, but, hopefully it hasn’t been too obvious to them.”* |
| **Behavioural/lifestyle interruptions** | *Reductions in activities of daily living* | *“I don’t work anymore. I haven’t gone back to work...financially it’s [my life] changed a lot, because I was on a good wage for the hours that I was doing.”* |
| **Psychological ill-being** | *Worry and uncertainty about the future* | *“Every year, even after this treatment is finished, I will go back to the hospital and they will tell me whether I need further treatment for this disease or not...So, the spell of living forever has been broken forever. You don’t know what your outcome’s going to be.”* |
|  | *Increased negative affect* | *It’s the unknown and just feeling miserable about what's going to happen [surgery], whether you're going to end up with a bag and things like that.”* |
|  |  |  |
| **Effects of exercise on HRQoL (n=13)** | | |
| **Physical well-being** | *Improved perceptions of strength and fitness* | *“So the exercise programme kept me fit. It kept me up to the standards where I was before. The chemo knocked me back a bit but the exercises then got me back on an even keel again, back on my bike again, so life’s carrying on as normal now.”* |
| **Psychological well-being** | *Improved positive affect* | *“I do feel better in myself, without a doubt. So, there’s definitely an improvement with the exercise...It’s [exercise] only made me more positive, because I feel better about myself.”* |
|  | *Decreased negative affect* | *“My perception of being ill has changed because I think if I didn’t take part in the course [exercise programme], I’d have gone home, said I’ve got cancer, sat there and worried myself to death...Coming to see you three times a week, got me out of the house and reassured me for any worries that I had.”* |
|  | *Manage disease and treatment-related concerns* | *“It’s [exercise training] strengthened my body for what's coming next - some man using a sharp implement on me... I would probably be more nervous about tomorrow than I am now. I think doing it [exercise training], I'm more confident going into tomorrow than I would have been if I hadn't done it. The exercise and preparation that I've been put through has got me all ready for it. I just want to get it over and done with.”* |

Abbreviation: HRQoL: health related quality of life

**Supplementary Appendix 6 Table 4. Post-Operative Morbidity Scores**

|  | **Exercise** | | | | **Control** | | | |
| --- | --- | --- | --- | --- | --- | --- | --- | --- |
| **Sub scales** | **Number of days post-surgery (n=13)** | | | | **Number of days post-surgery (n=8)** | | | |
|  | **3 (n=13)** | **5 (n=13)** | **8**  **(n=8)** | **15 (n=4)** | **3 (n=8)** | **5 (n=7)** | **8 (n=6)** | **15 (n=2)** |
| Pulmonary | 4 (33%) | 3 (25%) | 1 (13%) | 0 (0%) | 1 (13%) | 0 (0%) | 0 (0%) | 0 (0%) |
| Infectious | 3 (25%) | 5 (42%) | 3 (38%) | 2 (50%) | 2 (25%) | 2 (29%) | 1 (17%) | 1 (50%) |
| Renal | 9 (75%) | 4 (33%) | 4 (50%) | 1 (25%) | 5 (63%) | 4 (57%) | 3 (50%) | 0 (0%) |
| Gastrointestinal | 6 (50%) | 3 (25%) | 4 (50%) | 1 (25%) | 3 (38%) | 4 (57%) | 3 (50%) | 1 (50%) |
| Cardiovascular | 0 (0%) | 0 (0%) | 1 (13%) | 0 (0%) | 1 (13%) | 1 (14%) | 0 (0%) | 0 (0%) |
| Neurological | 2 (17%) | 1 (8%) | 0 (0%) | 0 (0%) | 1 (13%) | 0 (0%) | 0 (0%) | 0 (0%) |
| Hematological | 0 (0%) | 0 (0%) | 0 (0%) | 0 (0%) | 0 (0%) | 0 (0%) | 0 (0%) | 0 (0%) |
| Wound | 0 (0%) | 1 (8%) | 0 (0%) | 0 (0%) | 0 (0%) | 0 (0%) | 0 (0%) | 1 (50%) |
| Pain | 4 (33%) | 3 (25%) | 1 (13%) | 1 (25%) | 6 (75%) | 3 (43%) | 1 (17%) | 0 (0%) |

Data are presented as n (%).

The sub scales represent nine domains of post-operative morbidity.

**Supplementary Appendix 7 Table 5. Summary of response to treatment and histopathology**

| **Response** | **Exercise (n=15)** | | **Control (n=13)** | |
| --- | --- | --- | --- | --- |
| *yp_TRG* | **n** | **%** | **n** | **%** |
| 0 | 2 | 13 | 1 | 8 |
| 1 | 3 | 20 | 3 | 23 |
| 2 | 2 | 13 | 4 | 30 |
| 3 | 4 | 27 | 1 | 8 |
| 4 | 4 | 27 | 4 | 31 |
|  |  |  |  |  |
| *ymr_TRG* |  |  |  |  |
| 1 | 0 | 15 | 1 | 8 |
| 2 | 2 | 15 | 3 | 23 |
| 3 | 7 | 31 | 5 | 38 |
| 4 | 5 | 31 | 4 | 31 |
| 5 | 0 | 0 | 0 | 0 |
| Missing | 1 | 8 | 0 | 0 |
|  |  |  |  |  |
|  | **Exercise (n=13)** | | **Control (n=8)** | |
| **Histopathology** | **n** | **%** | **n** | **%** |
| *Histology T* |  |  |  |  |
| T0 | 3 | 23 | 1 | 12 |
| T1 | 1 | 8 | 3 | 38 |
| T2 | 1 | 8 | 1 | 12 |
| T3 or T3a | 8 | 61 | 3 | 38 |
| *Histology N* |  |  |  |  |
| N0 | 12 | 92 | 8 | 100 |
| N1 | 1 | 8 | 0 | 0 |
| *Histology R* |  |  |  |  |
| R0 | 12 | 92 | 8 | 100 |
| R1 | 0 | 0 | 0 | 0 |
| Missing | 1 | 8 | 0 | 0 |

Abbreviations: ypTRG (histopathological tumour regression grading); ymrTRG (MR tumour regression grading).

Response for ypTRG graded as follows: ypTRG 0 (no regression); ypTRG 1 (dominant tumour cell mass (>50 %) with obvious fibrosis or no regression); ypTRG 2 (dominantly fibrotic changes with few tumour cells or groups); ypTRG 3 (very few tumour cells (one or two miscoscopic foci of <0.5cm in diameter); ypTRG 4 (no tumour cells).

Response for ymrTRG graded as follows: ymrTRG 1 – 2 (favourable) and ymrTRG 3-5 (unfavourable).
